# Dietary Fermented Rapeseed Meal During the Grower Period Affects Growth Performance, Intestinal Health, and Antioxidant Status in *Sansui* Ducks

**DOI:** 10.3390/ani15142078

**Published:** 2025-07-14

**Authors:** Yulong Feng, Meijuan Li, Yuxi Lu, Chengcheng Tian, Yu Zhao, Jianwei Li, Zhiguo Wen, Yongwen Zhu

**Affiliations:** 1 Institute of Animal Husbandry and Veterinary Medicine, Guizhou Academy of Agricultural Sciences, Guiyang 550005, China; yulonfeng@126.com (Y.F.); 18286107320@163.com (C.T.); zhaoyu@gzsnky.wecom.work (Y.Z.); 2 Guizhou Testing Center for Livestock and Poultry Germplasm, Guiyang 550018, China; j15085907068@126.com; 3 Key Laboratory of Feed Biotechnology of Ministry of Agriculture, Institute of Feed Research, Chinese Academy of Agricultural Sciences, Beijing 100081, China; 4 Guangdong Provincial Key Laboratory of Animal Nutrition and Regulation, College of Animal Science, South China Agricultural University, Guangzhou 510000, China

**Keywords:** fermented rapeseed meal, duck, growth performance, intestinal health

## Abstract

The aim was to investigate effects of various proportions (0 to 20%) of fermented rapeseed meal (FRSM) on performance, intestinal health, and antioxidant status in *Sansui* ducks (*Sansui Sheldrake* ducks). A total of 350 male ducklings, 15 d old, were allocated into five dietary treatments with seven replicate pens of 10 birds per pen. The starter diets had 0, 5, 10, 15, or 20% FRSM that contained 0, 1.08, 2.17, 3.25, or 4.34 µmol glucosinolates (GLS/g of complete feed, respectively, fed from d 15 to d 35. The average daily feed intake (ADFI) and feed conversion ratio (FCR) from d 15 to 35 increased linearly with the dietary FRSM inclusion, with the ducks fed 15 or 20% FRSM diets having higher (*p* < 0.05) ADFIs and FCRs than the ducks fed 0% FRSM. The dietary FRSM inclusion levels of up to 15% were accompanied by decreased catalase (CAT) and antioxidant capacity (AOC) activities in the livers and jejunum of the ducks at d 35. In addition, the 20% FRSM (4.34 µmol GLS/g diet) decreased the villus height, crypt depth, muscular thickness, and goblet cell counts in the jejunum and ileum. In conclusion, 15 or 20% dietary FRSM (3.25 or 4.34 µmol GLS/g diet) during the grower period in *Sansui* ducks decreased feed efficiency, disrupted hepatic antioxidant balance, and impaired intestinal morphology and structure. The FRSM inclusion of ≥15% (3.25 µmol glucosinolates/g) impaired feed efficiency and antioxidant activity, while the 20% FRSM (4.34 µmol glucosinolates/g) induced intestinal damage in the growing *Sansui* ducks.

## Simple Summary

Rapeseed meal as the byproduct of the oil extraction of rapeseed can be used as a low-cost protein feed for farm animals. However, rapeseed meal contains natural compounds (glucosinolates) that can impair the growth rates and intestinal functions of ducks if used in excess. While small amounts are safe in complete feed, high levels may damage health. Here, we tested whether fermentation could allow higher inclusion of rapeseed meal without increasing risks for growing ducks. We fed young *Sansui* ducks five different diets containing 0%, 5%, 10%, 15%, or 20% fermented rapeseed meal for three weeks and monitored their growth, gut health, and resistance to stress. Our findings showed that the ducks given higher amounts (15–20%) of fermented rapeseed meal ate more but converted the feed into body weight less efficiently. Additionally, these ducks had weaker antioxidant defenses in their liver and intestines, along with thinner gut walls and fewer protective cells. This research will help farmers use fermented rapeseed meal more safely. Small amounts (up to 10%) appear to be a viable option, but exceeding this level may reduce duck growth and well-being.

## 1. Introduction

Due to shortages of conventional feeds and soaring prices for soybean meal, unconventional protein feedstuffs as alternative protein sources have been studied for poultry feed formulation to reduce feed costs [1]. To successfully use unconventional feedstuffs in animal diets, e.g., oilseed meals as a byproduct of oil extraction processing, such as rapeseed meal and cottonseed meal, were essential to evaluate nutritional composition, amino acid profiles, digestibility, and anti-nutritional factors [2]. Rapeseed meal (RSM) is an alternative protein source that can be used widely as a supplement or a replacement for traditional soybean meal in poultry diets. Including 150 to 200 g/kg RSM in diets has had no apparent negative effects on performance or health in broilers [3], turkeys [4,5], or ducks [6]. However, the use of RSM in poultry diets has been mainly limited by the presence of anti-nutritional factors, such as glucosinolates (GLS) and sinapine [7], as RSM with excessive anti-nutritional factors can reduce performance by damaging the thyroid, liver [6], or gut [8]. Therefore, with careful consideration of its anti-nutritional factors, it is important to determine appropriate levels of RSM to maximize benefits and minimize potential negative effects on poultry health and performance.

Modern varieties of double-low rapeseed (erucic acid < 20 g/kg and GLS < 30 µmol/g) and appropriate processing methods have reduced concentrations of anti-nutritional factors [9]. For traditional feedstuffs, fermentation is regarded as the most cost-effective way to increase the potential use of a feed product for poultry, including improved nutrient digestibility and reduced anti-nutritional factors [10]. Furthermore, microbial proteins produced from fermented RSM can be high-quality protein sources for poultry [11]. In addition to the type of RSM and concentrations of anti-nutritional factors, the effectiveness of RSM usage also depends on the breed of poultry [12,13]. For example, broiler strains at the same age have been more sensitive to the anti-nutritional factors of RSM than layer strains, as determined by increased trimethylamine oxidase activity in the liver [13].

Fermented RSM (FRSM) as a sustainable alternative to traditional protein sources has been used extensively to reduce the feed costs of duck production in China. Compared with fast-growing duck types (*Peking* duck and *Cherry Valley* duck) [6,14], Chinese native duck types (local Sheldrake) have slower growth rates and appear to have more digestive capability for RSM. It has been speculated that the tolerance of anti-nutritional factors from RSM inclusion in ducks could differ between fast- and slow-growing duck breeds.

Therefore, our objective was to determine the effect of inclusion levels of FRSM (0 to 20%) on performance, carcass characteristics, intestinal morphology, and antioxidant ability in Chinese native ducks (*Sansui Sheldrake* ducks) from 15 to 35 d of age.

## 2. Materials and Methods

### 2.1. Preparation of Fermented RSM

The RSM was obtained from Guizhou Youyan Chunxiang Ecological Grain and Oil Technology Co., Ltd. (Guiyang, China). The RSM contained (dry matter basis) 19.74 MJ/kg gross energy (GE), 35.64% crude protein (CP), 13.32% crude fiber (CF), and 47.58 μmol/g glucosinolates (GLS). The GE was determined by an Oxygen Bomb Calorimeter (Parr 6100; Parr, Moline, IL, USA). The CP was calculated from sample nitrogen content determined using the Dumas combustion method (AOAC [15]). The CF was determined according to the procedure described by the National Standards Committee [16]. The GLS content in the RSM was determined by isocratic liquid chromatography, as described [17]. The apparent metabolic energy (AME, 15.59 MJ/kg) and true metabolic energy (TME, 15.82 MJ/kg) of the *Sansui* ducks were measured by the emptying–force feeding method, as reported by Wei et al. [18].

The RSM was fermented as described below. The RSM was mixed with glucose powder (purity, 99%) at a mass ratio of 100:1, then inoculated with 7% fermentation bacteria (*Lactococcus lactis* ACCC10637, *Bacillus natto* CGMCC1.1086, and *Saccharomyces cerevisiae* CICC31011; mass ratio of 1:1:1); sterile water was added until the ratio of material to water was 1:1.1, and then it was placed in a bed-packed incubator for anaerobic fermentation at 37 °C for 48 h to obtain FRSM. The fresh fermented samples were dried in a hot-air oven at 80 °C for 3 d. The FRSM had analyzed nutritive values (dry matter basis) of 18.92 MJ/kg GE, 36.39% CP, 12.56% CF, 21.69 μmol/g GLS, 14.64 MJ/kg AME, and 14.87 MJ/kg TME for the *Sansui* ducks.

### 2.2. Birds, Management, and Diets

All experimental procedures were reviewed and approved by the Institute of Animal Husbandry and Veterinary Medicine, Guizhou Academy of Agricultural Sciences. A total of 350 *Sansui* ducklings, 1-day-old males, were kept in floor pens in a building with central heating and maintained at 34 °C from 1 to 3 d of age, which was decreased to 25 °C in decrements of 1 °C/day and thereafter kept constant (24–26 °C). All ducklings were fed a corn–soybean meal diet (11.72 MJ/kg AME, 195.5 g/kg CP, 9.5 g/kg lysine, 4.6 g/kg methionine, 8.6 g/kg calcium, 3.9 g/kg non-phytate phosphorus) for 14 d. At 15 d of age, the birds were weighed individually and allocated into 5 dietary treatments with 7 replicate pens of 10 birds per pen. During the experimental period, from d 15 to 35, the ducks were fed experimental diets (corn–soybean meal basal diet) with inclusion levels of 0, 5, 10, 15, or 20% FRSM that contained 0, 1.08, 2.17, 3.25, or 4.34 µmol GLS/g diets based on the GLS content of the FRSM. All experimental diets were isoenergetic and isonitrogenous and were formulated to meet or exceed the NRC [19] nutrient requirements of ducks in the grower–finisher period (Table 1). The experimental diets and water were provided ad libitum. The ducks’ care and management were in accordance with guidelines approved by the Chinese native duck farm.

### 2.3. Sample Collection

After 12 h of feed withdrawal at 35 d of age, the ducks were weighed and the feed consumption was monitored by each replicate pen for the calculation of the average daily gain (ADG), average daily feed intake (ADFI), and gain:feed ratio (FCR). At 35 d of age, a total of 8 ducks (closest to the average BW of the pen) were selected and euthanized by CO_2_ inhalation and then immediately bled. The liver, spleen, gizzard, breast, and thigh muscle were removed and weighed. The percentages of the breast and thigh muscle were expressed as percentages of the live body weight. The abdominal fat included fat tissues surrounding the proventriculus and gizzard, lying against the inside abdominal wall, and around the cloaca. The liver, spleen, and gizzard were expressed as percentages of the live body weight. Samples of the liver and jejunum were collected, snap-frozen in liquid N_2_, and stored at −80 °C for further analyses. Segments of the jejunum and ileum were flushed with physiological saline to remove all contents and fixed in 4% buffered paraformaldehyde.

### 2.4. Sample Preparation and Analyses

The liver and jejunum samples were homogenized in 10% (*w*/*v*) physiological saline on ice for 60 s and then sonicated with an ultrasonic wave cell grinder (JY92-11; NingBo Scientz Biotechnology Company, Ningbo, China) for 1 min (on 1 s; interval, 2 s). The homogenates were centrifuged at 1000× *g* for 15 min at 4 °C, the supernatants were collected to determine the total protein contents, and the indices were related to the antioxidant capacity and immune status. The total protein concentration was determined using a BCA Protein Assay kit (cat no. 23225; Pierce, Kyotom, Japan). The activities of glutathione peroxidase (GSH-Px), superoxide dismutase (SOD), and catalase (CAT); the total antioxidant capacity (T-AOC); and the malondialdehyde (MDA) content in the liver and jejunum were analyzed according to the instructions of the reagent kits (Nanjing Jiancheng Bioengineering Institute, Nanjing, China).

The concentrations of secretory immunoglobulin A (sIgA), interleukin (IL)-10, IL-6, IL-1β, and tumor necrosis factor α (TNF-α) in the jejunum were analyzed according to the instructions of the reagent kits (Shanghai Enzyme-linked Biotechnology Co., Ltd.; Shanghai, China). The total protein contents in the liver and jejunum were determined by the BCA Protein Assay Kit (Thermo, Rockford, IL, USA). The indices related to antioxidant capacity and immune status were expressed as U/mg protein.

Three cross-sections for the jejunum and ileum samples were prepared after staining with hematoxylin and eosin using standard paraffin embedding procedures. The eight longest intact villi from each section were selected for morphology measurement. The evaluated morphometric indices included villus height (VH), crypt depth (CD), villus height-to-crypt depth ratio (VH/CD), muscular thickness (MT), goblet cell count (GC), and goblet cell density (GD). Morphological indices were measured using an image processing and analysis system (Version 1, Leica Imaging System Ltd., Cambridge, UK).

### 2.5. Statistical Analyses

All values were subjected to one-way ANOVA by using the General Linear Model procedure of SAS 9.0 [20], and treatment comparisons were performed with the Duncan method. Orthogonal polynomials were applied for the linear and quadratic effects of the dependent variables to the independent variables. Each replicate served as an experimental unit for all statistical analyses. Significant differences were set at *p* ≤ 0.05.

## 3. Results

### 3.1. Growth Performance and Slaughter Characteristics

The dietary FRSM levels had no effects (*p >* 0.05) on the final BWs or ADGs from d 15 to 35 (Table 2) or the carcass traits at 35 d (Table 3). However, the ADFIs and FCRs increased (*p <* 0.05) linearly as the dietary FRSM levels increased (Table 2), with the ducks fed 15 or 20% FRSM diets having higher (*p <* 0.05) ADFIs and FCRs than the ducks fed 0% FRSM.

### 3.2. Intestinal Morphology

The dietary FRSM levels had no effects (*p >* 0.05) on the VH/CD and GD in the jejunum and ileum but affected (*p <* 0.05) other indices of intestinal morphology in the jejuna and ilea of the ducks at d 35 (Table 4 and Table 5). The ducks fed 20% FRSM had lower VHs, CDs, MTs, and GCs per unit of villi in the jejunum and ileum than other groups.

### 3.3. Immune Status in the Jejunum

The dietary FRSM levels had no effects (*p >* 0.05) on the IL-10, IL-6, IL-1β, or TNF-α contents but affected (*p <* 0.05) the sIgA content in the jejuna of the ducks at d 35 (Table 6); the ducks fed 0% FRSM had lower sIgA contents in the jejunum than those fed 5 to 20% FRSM.

### 3.4. Antioxidant Status in Liver and Jejunum

The dietary FRSM levels had no effects (*p >* 0.05) on the GSH-Px activity in the jejunum or the MDA content in the livers and jejunum of the ducks at d 35 but affected (*p <* 0.05) other indices in the liver and jejunum (Table 7 and Table 8). As the dietary FRSM levels increased, there were linear decreases in the activities of the CAT and AOC in the liver and jejunum and a linear increase in the SOD activity in the jejunum, plus a quadratic increase in the GSH-Px activity in the liver. The ducks fed 20% FRSM had higher SOD activities in the jejunum than those fed 0% FRSM and lower CAT and T-AOC activities in the jejunum than those fed 0 or 5% FRSM. The ducks fed 15 or 20% FRSM had lower activities of CAT, SOD, and T-AOC in the liver than the birds fed 0 or 5% FRSM. Finally, the ducks fed 0% FRSM had lower GSH-Px activity in the liver than the birds fed 5 to 15% FRSM.

## 4. Discussion

To reduce feed costs in China, RSM is widely used to replace soybean meal in poultry diets [1], with the proportion replaced limited by the presence of anti-nutritional factors, including GLS, sinapine, and non-starch polysaccharides (NSPs) [21]. In the present study, the fermentation reduced the GLS concentration from 47.58 to 21.69 μmol/g in the RSM, making it more suitable for inclusion in poultry feed. During fermentation, microorganisms such as lactobacilli, yeast, or bacilli can produce enzymes that hydrolyze GLSs into less harmful compounds [22]. Therefore, it was implied that the fermentation reduced the anti-nutritional effects of interference with nutrient absorption in poultry and enhanced the nutritional value of the RSM [23].

The dietary FRSM concentration fed during d 15–35 had no significant effect on the final BWs or ADGs in the Sansui ducks, consistent with studies replacing soybean meal with FRSM during d 15–45 in *Cherry Valley* ducks [24] and during d 1–42 in broilers [25]. However, the growth rate was depressed when the level of GLS increased to 6–10 µmol/g feed and was severely inhibited when it was >10 µmol/g feed [26]. Feeding up to 13.25% RSM (7.57 µmol/g GLS) had a negative effect on the growth performance of *Peking* ducks at d 15–35 [14].

In the present study, there was no growth depression in the ducks fed up to 20% FRSM (4.34 µmol/g GLS). Regardless, as the FRSM inclusion levels increased, both the ADFIs and FCRs were linearly increased in the *Sansui* ducks. Compared with the FCR of the ducks fed the control diet, the FCRs of the ducks fed diets with 15% FRSM (3.25 µmol GLS/g) or 20% FRSM (4.34 µmol GLS/g) were increased by 0.14 and 0.18, respectively. We inferred that local slow-growth ducks have a greater accepted tolerance of GLS, and they appeared to tolerate a higher incorporation rate of RSM in the diet than modern, fast-growth ducks.

The decreased feed efficiency could be partially due to the increased intake of anti-nutritional factors such as GLS, phytates, and NSPs as the FRSM increased, disrupting digestion and nutrient absorption and consequently reducing growth performance [27]. The available energy levels in the RSM were decreased as its first limiting factor (GLS level) was increased [28]. Although the primary anti-nutritional factor was decreased by fermentation, fermentation-derived metabolites (e.g., short-chain fatty acids, bacteriocins) might modulate nutrient availability in broilers and ducks [24,25]. In the present study, *Lactococcus lactis*, *Bacillus natto*, and *Saccharomyces cerevisiae* (mass ratio of 1:1:1) were used to produce organic acids and bioactive peptides that improved the intestinal health and then increased the nutrient digestibility of the ducks during the growing period. Additionally, in previous studies, the impaired performance of birds was related to the decreased protein digestibility and availability of amino acids in RSM, with increasing proportions of FRSM replacing soybean meal [29].

The GLS content and related hydrolysis products were the primary reason restricting the use of RSM and associated with damage to various tissues [30]. In this study, the intestinal morphology and structure were impaired in the ducks fed 20% FRSM, with decreased villus height and crypt depth as well as villus height-to-crypt depth ratios in the jejunum and ileum. Therefore, we inferred that the 20% FRSM had toxic effects of GLSs on intestinal health. Presumably, the impairment from the GLS reduced the intestinal absorption of nutrients, decreased the feed efficiency, and caused growth depression. Similar results have been confirmed in broilers [31]. When the GLS intake has exceeded the tolerance threshold, it induced severe tissue damage in ducks fed RSM [6] and also in broilers given grower diets with up to 24% RSM [32]. In addition, the damage to the intestinal morphology and structure was accompanied by an enhanced intestinal immune inflammatory response, decreased goblet cell density and proliferation per unit of villi, and increased sIgA content in the jejunum. However, there were no significant changes in the IL10, IL-6, IL-1β, or TNF-α in the jejuna of the ducks at 35 d, which was not consistent with broilers [31] or pigs [33].

Several studies have demonstrated that anti-nutritional factors potentially have a negative effect on antioxidant capacities in poultry [21]. In the present study, the dietary FRSM of >15% (3.25 µmol GLS/g diet) decreased the activities of CAT and T-AOC in the jejunum and liver. It has been speculated that GLS and hydrolysis products could interfere with the absorption of minerals and vitamins with antioxidant properties, thus reducing the overall antioxidant capacity [34]. For example, the hydrolysis products of GLS (e.g., goitrin) from RSM have impaired the uptake of iodide and iodide binding to the thyroglobulin of the thyroid and also influenced antioxidant status [35]. However, a high-vitamin premix supplementation prevented the negative effects of a 5 or 10% RSM diet in ducks by improving antioxidative capacities and alleviating liver and thyroid damage [34]. In the present study, as the dietary RSM inclusion level increased, the SOD activity increased linearly in the jejuna, whereas it decreased linearly in the livers, of the ducks at 35 d of age. The divergent responses in the SOD activity between the jejunum and liver implied tissue specificity.

## 5. Conclusions

In summary, up to 15% FRSM (3.25 µmol GLS/g diet) did not negatively affect growth but decreased feed efficiency and disrupted antioxidant activity in *Sansui* ducks from 15 to 35 d of age. In addition, 20% FRSM (4.34 µmol GLS/g diet) impaired the intestinal morphology and structure in the ducks at d 35. To make rational use of <15% FRSM in the diet, it is important to reduce potential negative impacts of anti-nutritional factors on intestinal health and antioxidant status in *Sansui* ducks.

## Figures and Tables

**Table 1:** Composition and nutrient concentrations (as-fed basis) in experimental diets during the grower period of Sansui ducks.

Ingredient (%)	Dietary Fermented Rapeseed Meal Level (%)				
	0	5	10	15	20
Corn	58.21	57.43	56.51	54.50	52.03
Corn starch	4.26	3.31	2.48	2.54	3.06
Wheat bran	0.11	1.12	2.15	3.15	4.04
Soybean meal	32.75	28.45	24.16	20.10	16.17
Fermented rapeseed meal	0.00	5.00	10.00	15.00	20.00
NaCl	0.30	0.30	0.30	0.30	0.30
Dicalcium phosphate	1.62	1.53	1.50	1.42	1.39
Limestone	1.14	1.15	1.12	1.13	1.10
Vitamin and mineral premix ^1^	1.00	1.00	1.00	1.00	1.00
DL-Methionine	0.15	0.16	0.15	0.15	0.15
L-Lysine sulphate	0.05	0.14	0.22	0.30	0.38
Soybean oil	0.41	0.41	0.41	0.41	0.38
Total	100.00	100.00	100.00	100.00	100.00
Calculated Nutrient Level					
Metabolizable energy (MJ/kg ^2^)	12.20	12.20	12.20	12.20	12.20
Crude protein (%)	19.50	19.50	19.50	19.50	19.50
Calcium (%)	0.90	0.90	0.90	0.90	0.90
Non-phytate phosphorus (%)	0.40	0.40	0.40	0.40	0.40
Methionine (%)	0.46	0.46	0.46	0.46	0.46
Methionine + cysteine (%)	0.80	0.80	0.80	0.80	0.80
Lysine (%)	1.05	1.05	1.05	1.05	1.05
Glucosinolates (µmol/g ^3^)	0	1.08	2.17	3.25	4.34

^1^ Provided per kilogram of diet: Cu (CuSO_4_•5H_2_O), 10 mg; Fe (FeSO_4_•7H_2_O), 60 mg; Zn (ZnO), 60 mg; Mn (MnSO_4_•H_2_O), 80 mg; Se (NaSeO_3_), 0.2 mg; I (KI), 0.2 mg; choline chloride, 1000 mg; vitamin A (retinol acetate), 10,000 IU; vitamin D_3_ (cholcalciferol), 3000 IU; vitamin E (DL-α-tocopheryl acetate), 20 IU; vitamin K_3_ (menadione sodium bisulfate), 2 mg; thiamin (thiamin mononitrate), 2 mg; riboflavin, 8 mg; pyridoxine hydrochloride, 4 mg; cobalamin, 0.06 mg; calcium-D-pantothenate, 20 mg; nicotinic acid, 50 mg; folic acid, 1 mg; biotin, 0.2 mg. ^2^ Values were calculated according to the AME values of feedstuffs for ducks. ^3^ Calculated values based on the analyzed value of 21.69 µmol/g glucosinolates in RSM.

**Table 2:** Effects of dietary fermented rapeseed meal inclusion levels (from 15 to 35 d of age) on growth performance of *Sansui* ducks ^1^.

Item	Dietary Fermented Rapeseed Meal Level (%)					SEM	*p*-Value	Linear	Quadratic
	0	5	10	15	20				
BW, g	1136	1139	1105	1127	1141	9.42	0.7703	0.9864	0.3493
ADG, g/d/bird	45.93	45.90	44.36	45.31	46.01	0.45	0.7773	0.9015	0.3219
ADFI, g/d/bird	103.1 ^c^	107.0 ^bc^	104.7 ^bc^	108.2 ^ab^	111.6 ^a^	0.79	0.003	0.0004	0.3724
FCR, g/g	2.25 ^b^	2.33 ^ab^	2.37 ^ab^	2.39 ^a^	2.43 ^a^	0.02	0.0284	0.0017	0.5066

^a–c^ Within a row, means without a common superscript differed (*p* < 0.05). ^1^ Data represent the means of seven replicate pens (*n* = 7) of ten birds per pen. ADFI, average daily feed intake; ADG, average daily gain; BW, body weight; FCR, feed conversion ratio.

**Table 3:** Effects of dietary fermented rapeseed meal inclusion levels (from 15 to 35 d of age) on carcass traits of *Sansui* ducks at 35 d of age ^1^.

Item, %	Dietary Fermented Rapeseed Meal (%)					SEM	*p*-Value	Linear	Quadratic
	0	5	10	15	20				
Breast muscle rate	3.08	3.07	3.14	2.71	2.82	0.07	0.2726	0.0963	0.6879
Thigh muscle rate	11.26	11.19	11.25	11.45	11.09	0.13	0.9445	0.9333	0.7011
Abdominal fat rate	0.84	0.69	0.84	0.57	0.63	0.04	0.2016	0.0911	0.9618
Liver	2.68	2.37	2.37	2.4	2.37	0.04	0.1231	0.0613	0.1218
Spleen	0.14	0.15	0.14	0.12	0.13	0.01	0.7141	0.3893	0.7964
Gizzard	3.72	3.64	3.68	3.57	3.59	0.06	0.9596	0.5147	0.9226

^1^ Data represent the means of eight ducks being analyzed (*n* = 8).

**Table 4:** Effects of dietary fermented rapeseed meal inclusion levels (from 15 to 35 d of age) on jejunal morphology in *Sansui* ducks at 35 d of age ^1^.

Item	Dietary Fermented Rapeseed Meal (%)					SEM	*p*-Value	Linear	Quadratic
	0	5	10	15	20				
VH, μm	900.25 ^a^	1061.31 ^a^	1021.44 ^a^	931.15 ^a^	459.84 ^b^	48	<0.0001	0.0003	<0.0001
CD, μm	247.83 ^b^	298.15 ^a^	279.67 ^ba^	291.00 ^ba^	116.06 ^c^	12.78	<0.0001	<0.0001	<0.0001
MT, μm	318.19 ^b^	387.60 ^a^	362.53 ^a^	358.69 ^a^	138.64 ^c^	15.34	<0.0001	<0.0001	<0.0001
VH/CD	3.75	3.44	3.74	3.38	4.11	0.14	0.5023	0.5076	0.2395
GC/villus	46 ^a^	53 ^a^	43 ^a^	42 ^a^	24 ^b^	3	0.0115	0.0035	0.0528
GD/mm	53	49	41	45	51	2	0.3322	0.5569	0.063

^a, b, c^ Means within a row lacking a common superscript differ (*p* < 0.05). ^1^ Data represent the means of eight ducks being analyzed (*n* = 8). VH, villus height; CD, crypt depth; MT, muscular thickness; VH/CD, villus height-to-crypt depth ratio; GC, goblet cell count; GD, goblet cell density.

**Table 5:** Effects of dietary fermented rapeseed meal inclusion levels (from 15 to 35 d of age) on the morphologies of the ilea in *Sansui* ducks at 35 d of age ^1^.

Item	Dietary Fermented Rapeseed Meal Level, %					SEM	*p*-Value	Linear	Quadratic
	0	5	10	15	20				
VH, μm	693.10 ^a^	671.12 ^a^	671.06 ^a^	713.50 ^a^	294.67 ^b^	27.27	<0.0001	<0.0001	<0.0001
CD, μm	219.16 ^a^	232.30 ^a^	225.01 ^a^	244.06 ^a^	91.75 ^b^	9.88	<0.0001	<0.0001	<0.0001
MT, μm	348.57 ^b^	377.74 ^b^	363.93 ^b^	416.36 ^a^	152.46 ^c^	15.59	<0.0001	<0.0001	<0.0001
VH/CD	3.29	3	3.07	3.02	3.56	0.09	0.2793	0.3901	0.0549
GC/villus	37 ^a^	35 ^a^	40 ^a^	36 ^a^	16 ^b^	2	<0.0001	0.0002	0.0002
GD/mm	54	52	60	51	54	2	0.6095	0.9772	0.6009

^a, b, c^ Means within a row lacking a common superscript differ (*p* < 0.05). ^1^ Data represent the means of eight ducks being analyzed (*n* = 8). VH, villus height; CD, crypt depth; MT, muscular thickness; VH/CD, villus height-to-crypt depth ratio; GC, goblet cell count; GD, goblet cell density.

**Table 6:** Effects of dietary fermented rapeseed meal inclusion levels (from 15 to 35 d of age) on the immune statuses of the jejuna of *Sansui* ducks at 35 d of age ^1^.

Item, pg/mg Protein	Dietary Fermented Rapeseed Meal Level, %					SEM	*p*-Value	Linear	Quadratic
	0	5	10	15	20				
IL-1β	55.93	48.67	47.53	54.51	54.4	1.85	0.5116	0.8343	0.1604
IL-10	23.51	27.93	27.15	30.44	30.09	0.92	0.1093	0.0156	0.4576
TNF-α	292.62	263.89	262.48	276.73	258.65	7.93	0.6711	0.3425	0.589
IL-6	9.33	7.16	8.11	9.41	8.59	0.33	0.1644	0.7278	0.2568
sIgA	13,502 ^b^	16,535 ^ba^	17,965 ^a^	19,980 ^a^	18,228 ^a^	712	0.0448	0.0087	0.1107

^a, b^ Means within a row lacking a common superscript differ (*p* < 0.05). ^1^ Data represent the means of eight ducks being analyzed (*n* = 8). IL-1β, interleukin 1β; IL-10, interleukin 10; TNF-α, tumor necrosis factor α; IL-6, interleukin 6; sIgA, secretory immunoglobulin A.

**Table 7:** Effects of dietary fermented rapeseed meal inclusion levels (from 15 to 35 d of age) on the antioxidant statuses of the livers of *Sansui* ducks at 35 d of age ^1^.

Item	Dietary Fermented Rapeseed Meal Level, %					SEM	*p*-Value	Linear	Quadratic
	0	5	10	15	20				
CAT, U/mg protein	92.06 ^a^	80.33 ^abc^	85.34 ^ab^	65.14 ^c^	72.2 ^bc^	2.92	0.0225	0.0056	0.5773
GSH-Px, U/g protein	171.47 ^b^	245.21 ^a^	232.82 ^a^	259.39 ^a^	209.57 ^ab^	8.44	0.0047	0.0859	0.0015
SOD, U/mg protein	107.09 ^a^	90.78 ^a^	53.07 ^b^	30.69 ^b^	28.28 ^b^	5.75	<0.0001	<0.0001	0.3067
T-AOC, μmol Trolox/g	411.16 ^a^	365.07 ^a^	368.21 ^a^	294.83 ^b^	299.08 ^b^	11.43	0.0001	<0.0001	0.7613
MDA, nmol/mg protein	11.77	9.52	9.88	9.65	9.37	0.44	0.4062	0.1381	0.3644

^a, b, c^ Means within a row lacking a common superscript differ (*p* < 0.05). ^1^ Data represent the means of eight ducks being analyzed (*n* = 8). CAT, catalase; GSH-Px, glutathione peroxidase; MDA, malondialdehyde; SOD, superoxide dismutase; T-AOC, total antioxidant capacity.

**Table 8:** Effects of dietary fermented rapeseed meal inclusion levels (from 15 to 35 d of age) on the antioxidant statuses of the jejunum of *Sansui* ducks at 35 d of age ^1^.

Item	Dietary Fermented Rapeseed Meal Level, %					SEM	*p*-Value	Linear	Quadratic
	0	5	10	15	20				
CAT, U/mg protein	82.95 ^ab^	89.71 ^a^	83.34 ^ab^	72.96 ^bc^	67.31 ^c^	2.20	0.0048	0.001	0.0765
GSH-Px, U/g protein	188.4 ^b^	248.0 ^ab^	228.8 ^ab^	276.0 ^a^	280.8 ^a^	12.8	0.1353	0.0198	0.6769
SOD, U/mg protein	36.79 ^c^	59.29 ^bc^	44.16 ^c^	75.77 ^ab^	90.47 ^a^	5.024	0.0011	0.0001	0.3717
T-AOC, μmol Trolox/g	420.3 ^a^	388.2 ^ab^	364.8 ^abc^	343.9 ^bc^	316.2 ^c^	11.1	0.0253	0.0011	0.8946
MDA, nmol/mg protein	14.11	10.15	11.98	13.31	11.24	0.56	0.1605	0.5007	0.4667

^a, b, c^ Means within a row lacking a common superscript differ (*p* < 0.05). ^1^ Data represent the means of eight ducks being analyzed (*n* = 8). CAT, catalase; GSH-Px, glutathione peroxidase; MDA, malondialdehyde; SOD, superoxide dismutase; T-AOC, total antioxidant capacity.

## Data Availability

The data presented in this study are available on request from the corresponding author on reasonable request.
